# Epidemiology, outcomes and risk factors for recurrence of *Clostridioides difficile* infections following allogeneic hematopoietic cell transplantation: a longitudinal retrospective multicenter study

**DOI:** 10.1038/s41409-023-02157-3

**Published:** 2023-11-30

**Authors:** Silvio Ragozzino, Nicolas J. Mueller, Dionysios Neofytos, Jakob Passweg, Antonia Müller, Michael Medinger, Christian Van Delden, Stavroula Masouridi-Levrat, Yves Chalandon, Sarah Tschudin-Sutter, Nina Khanna, Swiss Transplant Cohort Study, Christian Van Delden

**Affiliations:** 1 Division of Infectious Diseases and Hospital Epidemiology, University Hospital Basel, Basel, Switzerland; 2 Division of Infectious Diseases and Hospital Epidemiology, University Hospital Zurich, Zurich, Switzerland; 3 Transplant Infectious Diseases Unit, University Hospitals Geneva, Geneva, Switzerland; 4 Division of Hematology, University Hospital Basel, Basel, Switzerland; 5 Department of Medical Oncology and Hematology, University Hospital Zurich, Zurich, Switzerland; 6 Department of Transfusion Medicine and Cell Therapy, Medical University of Vienna, Vienna, Austria; 7 Division of Hematology, University Hospitals Geneva and Faculty of Medicine, University of Geneva, Geneva, Switzerland

**Keywords:** Infectious diseases, Risk factors

## To the Editor:

Hematopoietic cell transplant (HCT) recipients are at increased risk of developing *Clostridioides difficile* infection (CDI) due to their longer healthcare and antibiotic exposure, and immunocompromised status [1].

In HCT recipients, CDI seems to occur more frequently in the pre-engraftment period, although more recent evidence suggests that an important proportion of CDI develops during the late post-transplant period [2]. Most previous studies captured limited follow-up periods and clinical outcomes are poorly described.

Given the paucity of long-term surveillance and outcome data of CDI in allogeneic HCT recipients, we conducted a multicenter retrospective study within the Swiss Transplant Cohort Study, which includes all adult allogeneic HCT recipients in the three Swiss HCT centers over a nine-year period. Primary outcomes were CDI recurrence in the first 8 weeks after the index episode and the identification of recurrence risk factors. As secondary outcomes we describe: disease severity and development of complications; 30-day and 1-year all-cause mortality.

Between 2009 and 2018, 1540 allogeneic HCT were performed in Swiss centers with a median follow-up time of 3.7 years (interquartile range [IQR]: 1.0–5.5). Overall, 131 patients developed CDI accounting for an incidence of 8.5%. The number of allogeneic HCT performed, as well as the CDI episodes increased during the study period. The CDI rate showed a non-significant increasing trend over time (Fig. S1, supplementary material). Median time post-transplant to CDI was 61 days (IQR: 10–180). Relevant clinical characteristics of the included patients are shown in supplementary material (Tables S1, S2). Median age of the included patients was 51 years (IQR: 41–62) and 60.3% were male. Acute myeloid leukemia was the most frequent underlying disease (*n* = 58, 44.3%). Overall, 85.5% of the patients (*n* = 112) had received an antibiotic therapy in the 3 months prior CDI. Antibiotic prophylaxis was administered in 96.2% (*n* = 126) of patients (mostly trimethoprim/sulfamethoxazole as *Pneumocystis jirovecii* prophylaxis; five patients received quinolone prophylaxis) and 84.0% (*n* = 110) were treated with proton pump inhibitors. Most of the patients had received a chemotherapy regimen (*n* = 56, 42.7%) and/or other immunosuppressive therapy in the last month (*n* = 102, 77.9%).

CDI was classified as healthcare-associated in 98 patients (74.8%). The diagnostic strategy differed among centers and changed over the study period: in most of the cases (65.6%), the diagnosis was made with a two-step approach, mostly through the determination of the glutamate dehydrogenase antigen followed by the toxin detection by polymerase chain reaction (57.3%). Twenty-four patients (18.3%) had severe disease, defined by a Zar score ≥2, and six patients (4.0%) had complications such as ileus (*n* = 3), hypotension/shock (*n* = 3) and toxic megacolon (*n* = 1).

The most common therapy was oral metronidazole (*n* = 87, 66.4%). Monotherapy with oral vancomycin was used in 20 (15.3%) and fidaxomicin in 3 (2.3%) patients. One patient received fecal microbiota transplantation after the third recurrence.

Median follow-up after CDI onset was 2.9 years (IQR 0.6–4.8). Twenty-three patients (17.6%) had a recurrence in the 8 weeks after the primary CDI episode. Thirty-day mortality was 6.9% and 1-year mortality 23.7%.

Regarding timing of CDI diagnosis, CDI occurred in 46 cases (35.1%) in the peri-transplant period and in 85 cases (64.9%) in the post-engraftment period (25.2% in the early post-engraftment period [30–100 days after HCT] and 39.7% in the late post-engraftment period [>100 days after HCT]). No significant differences in clinical severity, 30-day mortality, and recurrence rate were observed according to the timing of CDI diagnosis (Table S3).

The results of the analysis of the risk factors for recurrence are shown in Table 1. In the multivariable analysis, the number of prior antibiotics (OR 1.54; 95% CI 1.12–2.12; *p* = 0.008), immunosuppressive therapy in the month prior to CDI diagnosis (OR 9.69; 95% CI 1.06–88.45; *p* = 0.04) and a malignancy other than acute leukemia (OR 4.85; 95% CI 1.64–14.32; *p* = 0.004) were independently associated with recurrence. One-year mortality was higher among patients with recurrence than without, without reaching statistical significance (36.4% vs. 19.8%, *p* = 0.10).

**Table 1:** Comparison between CDI patients without (non r-CDI) and with recurrent disease (r-CDI).

	Total (*n* = 118)	Non r-CDI (*n* = 96)	r-CDI (*n* = 22)	Univariable *P* value^a^	Multivariable^b^ OR (95% CI); *P*
Age (years), median (IQR)	51 (41–62)	50 (40–61)	57 (41–63)	0.36	NS
Male, *n* (%)	74 (62.7)	61 (63.5)	13 (59.1)	0.70	
Healthcare related, *n* (%)	89 (75.4)	69 (71.9)	20 (90.9)	0.06	NS
Prior antibiotic therapy, *n* (%)	99 (83.9)	79 (82.3)	20 (90.9)	0.52	
Number of antibiotics, median (IQR)	2 (1–4)	2 (1–3)	4 (1–4)	0.03	1.54 (1.12–2.12); P 0.008
β-Lactams/β-Lactamase inhibitors, *n* (%)	56 (47.5)	42 (43.8)	14 (63.6)	0.10	
Cephalosporin, *n* (%)	55 (46.6)	40 (41.7)	15 (68.2)	0.02	NS
Carbapenems, *n* (%)	45 (38.1)	34 (35.4)	11 (50.0)	0.20	
Quinolons, *n* (%)	26 (22.0)	21 (21.9)	5 (22.7)	0.93	
Proton pump inhibitors, *n* (%)	99 (83.9)	80 (83.3)	19 (86.4)	1.00	
Neutropenia, *n* (%)	34 (28.8)	30 (31.2)	4 (18.2)	0.22	
Immunosuppressive therapy, *n* (%)	91 (77.1)	70 (72.9)	21 (95.5)	0.02	9.69 (1.06–88.45); P 0.04
Chemotherapy, *n* (%)	48 (40.7)	41 (42.7)	7 (31.8)	0.47	
Malignancy other than acute leukemia, *n* (%)	44 (37.3)	30 (31.2)	14 (63.6)	0.005	4.85 (1.64–14.32); P 0.004
Myeloablative conditioning, *n* (%)	78 (66.1)	64 (66.7)	14 (63.6)	0.79	
Timing of CDI occurrence^c^, *n* (%)				0.56	
Peri-transplant	44 (37.3)	37 (38.5)	7 (31.8)		
Postengraftment	74 (62.7)	59 (61.5)	15 (68.2)		
Timely related intestinal GvHD^d^, *n* (%)	16 (13.6)	13 (13.5)	3 (13.6)	1.00	
Zar score, median (IQR)	1 (0–1)	1 (0–1)	1 (0-1)	0.38	
Severe CDI (Zar ≥ 2), *n* (%)	19 (16.1)	15 (15.6)	4 (18.2)	0.75	
CDI treatment, *n* (%)					
Metronidazole alone	84 (71.2)	68 (70.8)	16 (72.7)	0.86	
Vancomycin alone	17 (14.4)	12 (12.5)	5 (22.7)	0.31	
Need for antibiotics after CDI^e^, *n* (%)	72 (61.0)	59 (61.5)	13 (59.1)	0.84	

*r-CDI* recurrent *C. difficile* infection, *OR* odds ratio, *95% CI* 95% confidence interval, *IQR* interquantile range, *NS* non-significant, *GVHD* graft versus host disease.

^a^*χ*2 and Fisher exact test for categorical variables; Mann-Whitney U test for continuous variables.

^b^Logistic regression analysis: factors with *P* values ≤ 0.1 in univariable analyses, and variables previously associated with recurrence, were included in the multivariable model. The same results were obtained by stepwise logistic regression using stepwise forward and backward selection, as well as Akaike Information Criterion. The Hosmer-Lemeshow goodness-of-fit test revealed an insignificant p-value (*χ*2 = 6.03, *p* = 0.644) indicating adequate model fit.

^c^Timing of CDI occurrence, peritransplant: CDI diagnosis 10 days before-30 days after HCT; post-engraftment: CDI diagnosis >30 days after HCT.

^d^Intestinal GVHD occurring 30 days before until 8 weeks after CDI diagnosis. Diagnosis of intestinal GVHD was confirmed through histological examination of endoscopic biopsies.

^e^Systemic antibiotics other than CDI-therapy within 8 weeks after CDI index episode.

This multicenter study provides a comprehensive description of CDI in allogeneic HCT recipients in Switzerland. We found an overall CDI incidence of 8.5% which is in line with the data reported in a previous metaanalysis [1]. Similarly to this study, we observed an increasing trend over time, which could be partially attributed to the enhanced sensitivity of molecular diagnostics now widely employed in most centers [3]. In our cohort, with an extensive surveillance after HCT, the majority of CDI cases (64.9%) were diagnosed during the post-engraftment period. These findings challenge previous research that identified the pre-engraftment period as the highest risk phase [4–6] and underscore the importance of maintaining a high degree of clinical suspicion for CDI even in later phases following transplantation.

Our findings showed that over 80% of patients had mild disease, consistent with previous studies in onco-hematological patients [7]. The lower prevalence of hypervirulent ribotypes in Switzerland, such as ribotype 027 [3], could contribute to this finding. Around 18% of patients in our cohort experienced CDI recurrence. This result aligns with previous reports in allogeneic HCT recipients [8–10] and is consistent with rates among general hospitalized population [11]. Metronidazole was the most commonly used therapy, reflecting the prevailing practice during the study period, in line with international guidelines in place at the time. Future studies are needed to assess whether current first line therapies (i.e., vancomycin/fidaxomicin) result in lower recurrence rates in this patient group.

In our cohort, the number of prior antibiotics, immunosuppressive therapy before CDI diagnosis, and malignancy other than acute leukemia were independent risk factors for recurrence. Cumulative antibiotic exposure disrupts the intestinal microbiota, promoting *C. difficile* overgrowth and increasing recurrence risk. This aligns with a retrospective US study showing a twofold increase in recurrence risk with each additional antibiotic used [12]. The association of immunosuppressive therapy with recurrence is possibly due to a reduced immune response during the initial CDI episode. Reasons for higher recurrence risk in patients with malignancies other than acute leukemia remain unclear. They may undergo transplantation at advanced disease stages, receiving extensive pretreatment and experiencing severe alteration of the intestinal microbiota.

Limitations of our study include retrospective data collection, heterogeneity in diagnostic procedures, and a relatively small number of recurrent cases. Due to the small number of patients receiving antibiotic prophylaxis (apart from *P. jirovecii* prophylaxis), no analyses could be performed on the influence of antibiotic prophylaxis on the course of CDI. Data on cytomegalovirus screening was not available for all patients, so that an association between cytomegalovirus colitis and CDI could not be assessed. Our study design did not allow us to assess correlations between chronic graft-versus-host disease and CDI, and no data were available to categorize the timing of T-cell immune reconstitution.

In conclusion, our study highlights that CDI in allogeneic HCT recipients is most commonly observed during the post-engraftment period and is generally mild in nature in settings with low circulation of the hypervirulent ribotype 027. The recurrence rate is similar to the general population, but patients with recurrent disease show a trend for increased 1-year mortality. Risk factors for recurrence include prior antibiotic use, immunosuppressive therapy, and malignancy other than acute leukemia. This knowledge enables interventions to target modifiable risk factors such as antibiotic and immunosuppressive therapies. Identifying patients at higher risk for CDI recurrence would facilitate the selection of appropriate candidates for novel treatment strategies specifically designed to target recurrent CDI.

### Supplementary information

Supplementary Material

## Data Availability

The datasets analyzed for this study are available from the corresponding author upon specific request.
