# Supplementary material for: Epidemiology, outcomes and risk factors for recurrence of Clostridioides difficile infections following allogeneic hematopoietic cell transplantation: a longitudinal retrospective multicenter study
Source: Bone Marrow Transplant. 2023 Nov 30;59(2):278–81. doi: 10.1038/s41409-023-02157-3 (PMC10849940; doi:10.1038/s41409-023-02157-3)
Supplement: Supplementary file 1 — Supplementary Material [file 41409_2023_2157_MOESM1_ESM.docx]

**Supplementary material**

This study has been conducted in the framework of the Swiss Transplant Cohort Study, supported by the Swiss National Science Foundation, the Swiss University Hospitals, and transplant centers. The members of the Swiss Transplant Cohort Study are: Patrizia Amico, John-David Aubert, Adrian Bachofner, Vanessa Banz, Sonja Beckmann, Guido Beldi, Christoph Berger, Ekaterine Berishvili, Annalisa Berzigotti, Pierre-Yves Bochud, Sanda Branca, Heiner Bucher, Anne Cairoli, Emmanuelle Catana, Yves Chalandon, Sabina De Geest, Sophie De Seigneux, Michael Dickenmann, Joëlle Lynn Dreifuss, Michel Duchosal, Thomas Fehr, Sylvie Ferrari-Lacraz, Jaromil Frossard, Christian Garzoni, Déla Golshayan, Nicolas Goossens, Fadi Haidar, Jörg Halter, Dominik Heim, Christoph Hess, Sven Hillinger, Hans Hirsch, Patricia Hirt, Linard Hoessly, Günther Hofbauer, Uyen Huynh-Do, Franz Immer, Michael Koller, Andreas Kremer, Christian Kuhn, Bettina Laesser, Frédéric Lamoth, Roger Lehmann, Alexander Leichtle, Oriol Manuel, Hans-Peter Marti, Michele Martinelli, Valérie McLin, Katell Mellac, Aurélia Merçay, Karin Mettler, Nicolas Müller, Ulrike Müller-Arndt, Beat Müllhaupt, Mirjam Nägeli, Graziano Oldani, Manuel Pascual, Jakob Passweg, Rosemarie Pazeller, Klara Posfay-Barbe, David Reineke, Juliane Rick, Anne Rosselet, Simona Rossi, Rössler, Silvia Rothlin, Frank Ruschitzka, Thomas Schachtner, Stefan Schaub, Alexandra Scherrer, Dominik Schneidawind, Aurelia Schnyder, Macé Schuurmans, Simon Schwab, Thierry Sengstag, Federico Simonetta, Jürg Steiger, Guido Stirniman, Ueli Stürzinger, Christian Van Delden, Jean-Pierre Venetz, Jean Villard, Julien Vionnet, Madeleine Wick, Markus Wilhlem, Patrick Yerly.

**Supplementary figures and tables**

**Figure S1.** Temporal trends of the allogeneic HCT performed in the participating centers and CDI diagnosed over the study period.

Here we show the timeframe 2010-2017 (complete calendar year data collection). The grey pointed line shows an increasing trend in CDI incidence over recent years (Chi-square for linear trend p=0.09).

Allo-HCT: allogeneic hematopoietic cell transplantation; CDI: *C. difficile* infection

**Table S1**. Main baseline clinical features of the included patients (n=131)

| Age (years), median (IQR) | 51.0 (41-62) |
| --- | --- |
| Male, n (%) | 79 (60.3) |
| Oncohematological diagnosis, n (%)  Acute myeloid leukemia  Acute lymphoid leukemia  Myelodysplastic syndrome  Others | 58 (44.3)  22 (16.8)  15 (11.5)  36 (27.5) |
| HCT features, n (%)  Stem cell source  Peripheral blood  Bone marrow  Donor type  Matched unrelated  Matched related  Mismatched unrelated  Haplo-related  Conditioning regimen  Myeloablative  Reduced intensity  Non-myeloablative  Total body irradiation  GVHD prophylaxis  Cyclosporine A  Methotrexate  Anti-thymocyte globulin  Mycophenolate mofetil  Others | 122 (93.1)  9 (6.9)  58 (44.3)  49 (37.4)  19 (14.5)  5 (3.8)  86 (65.6)  26 (19.8)  19 (14.5)  53 (40.5)  119 (90.8)  77 (58.8)  57 (43.5)  45 (34.4)  12 (9.2) |
| Time to engraftment (days), median (IQR) | 16 (13-19) |
| Acute GVHD, n (%)  Intestinal GVHD | 76 (58.0)  44 (33.6) |
| Comorbidities, n (%)  Chronic lung disease  Diabetes mellitus  Charlson comorbidity index ≥3, n (%) | 20 (15.3)  15 (11.5)  59 (45.0) |

HCT: hematopoietic cell transplantation; IQR: interquartile range; GVHD: graft versus host disease.

**Table S2**. Main features of the CDI episodes: clinical presentation, management and outcomes (n=131)

| CDI classification, n (%)  Healthcare-associated  Community-acquired  Indeterminate/unknown | 98 (74.8)  14 (10.7)  19 (14.5) |
| --- | --- |
| Predisposing factors, n (%)  Prior CDI  Prior antibiotic therapy  β-Lactams/β-Lactamase inhibitors  Cephalosporins  Carbapenems  Quinolones  Proton-pump inhibitors  Neutropenia at CDI diagnosis  Immunosuppressive therapy  Chemotherapy | 17 (13.0)  112 (85.5)  64 (48.9)  60 (45.8)  49 (37.4)  31 (23.7)  110 (84.0)  39 (29.8)  102 (77.9)  56 (42.7) |
| CDI clinical presentation, n (%)  Severe (Zar score ≥2)  Complicated, n (%)  Rise in serum creatinine, n (%)  WBC >15 0000/µl, n (%)  Albumin <25g/L, n (%) | 24 (18.3)  6 (4.0)  13 (9.9)  10 (7.6)  30 (22.9) |
| CDI management, n (%)  Metronidazole  Vancomycin po  Others*  No therapy | 87 (66.4)  20 (15.3)  18 (13.7)  6 (4.6) |
| Need for antibiotic therapy after CDI diagnosis, n (%) | 80 (61.1) |
| CDI outcomes, n (%)  Bloodstream infections^&^ (≤30 days after index CDI)  Recurrence° (≤8 weeks after index CDI)  Reinfection° (>8 weeks after index CDI)  30-day mortality  1-year mortality | 7 (5.3)  23 (17.6)  14 (10.7)  9 (6.9)  31 (23.7) |

CDI: *Clostridioides difficile* infection; WBC: white blood cells.

*Other therapies: 9 patients treated with metronidazole (≥3 days) followed by vancomycin; 5 with vancomycin per os and intravenous metronidazole (± rectal vancomycin); 3 with Fidaxomicin; one with Linezolid.

^&^ Six out of the seven BSI were considered as possible mucosal barrier-associated infections.

° Two patients had one recurrence and one reinfection; one patient one recurrence and two reinfections; one patient one recurrence and three reinfections; one patient two recurrences and one reinfection; two patients two recurrences; one patient four recurrences; two patients had 2 reinfections. Overall, 32 patients (24.4%) had at least another CDI episode (recurrence or reinfection) after index case.


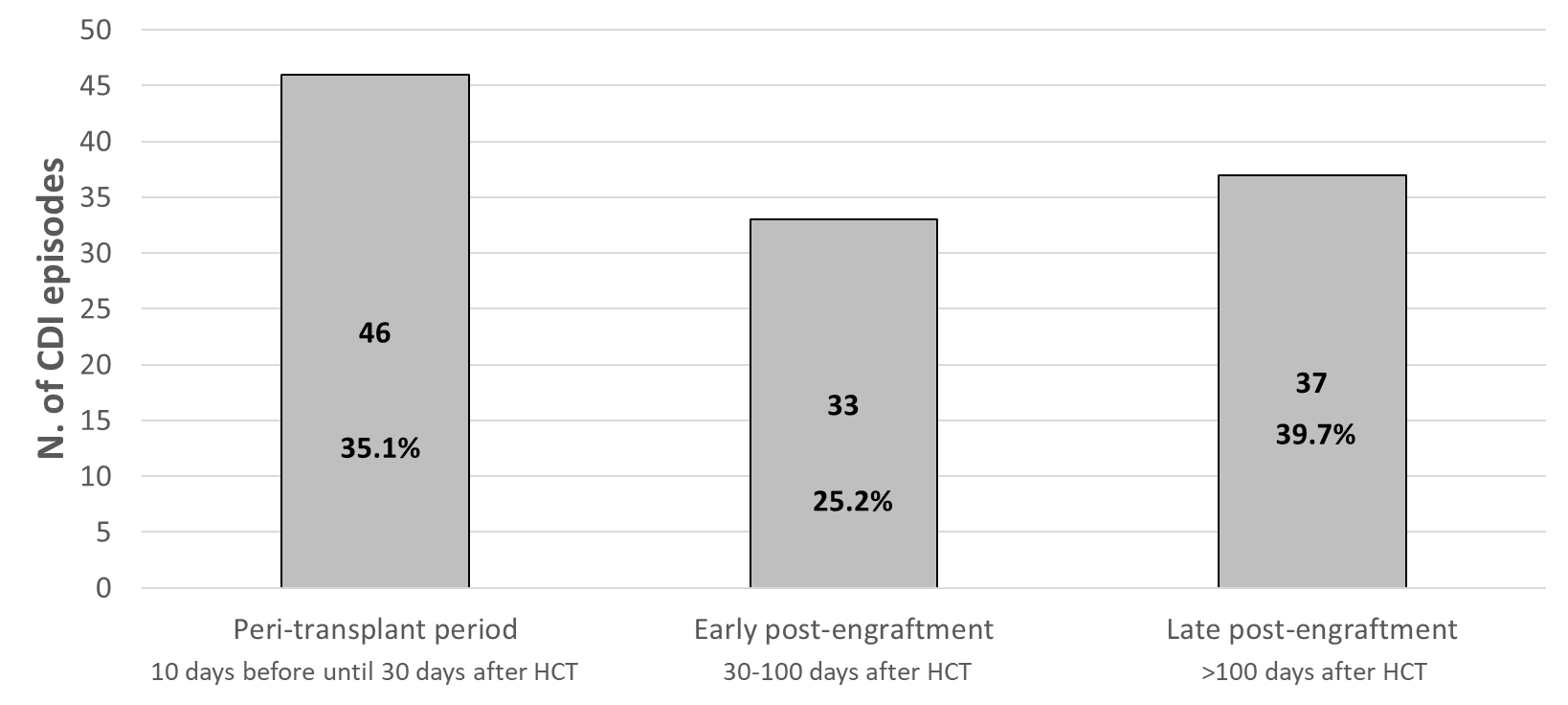


**Figure S2**. Timing of CDI occurrence in relation to allogeneic HCT

CDI: *C. difficile* infection, HCT: hematopoietic cell transplantation

**Table S3**. Comparison between CDI episodes occurring in the peri-transplant (n=79) and late post-transplant period (n=52)

|  | Peri-transplant* CDI (n=79) | Late post-transplant* CDI (n=52) | p-value |
| --- | --- | --- | --- |
| Age (years), median (IQR) | 56 (43-62) | 49 (38-63) | 0.24 |
| Male, n (%) | 46 (58.2) | 33 (63.5) | 0.55 |
| Healthcare related, n (%) | 65 (82.3) | 33 (63.5) | 0.02 |
| Charlson score, median (IQR) | 2 (2-3) | 2 (2-4) | 0.91 |
| Prior antibiotic therapy, n (%) | 74 (93.7) | 38 (73.1) | 0.001 |
| Proton pump inhibitors, n (%) | 70 (88.6) | 40 (76.9) | 0.07 |
| Neutropenia, n (%) | 32 (40.5) | 7 (13.5) | 0.001 |
| Immunosuppressive therapy, n (%) | 70 (88.6) | 32 (61.5) | <0.001 |
| Chemotherapy, n (%) | 48 (60.8) | 8 (15.4) | <0.001 |
| Zar score, median (IQR) | 1 (0-1) | 1 (0-1) | 0.86 |
| Severe CDI, n (%) | 13 (16.5) | 11 (21.2) | 0.50 |
| Complicated CDI, n (%) | 1 (1.3) | 5 (9.6) | 0.04 |
| Recurrence, n (%) | 15 (19.0) | 8 (15.4) | 0.60 |
| 30-day mortality, n (%) | 4 (5.1) | 5 (9.6) | 0.48 |
| 1-year mortality, n (%) | 19 (24.1) | 21 (40.4) | 0.05 |

CDI: *C. difficile* infection; HCT: hematopoietic cell transplantation; IQR: interquartile range.

* Peri-transplant CDI (including early post-engraftment): CDI diagnosis 10 days before-100 days after HCT; Late post-transplant CDI: CDI diagnosis >100 days after HCT.

14/85 patients with post-engraftment CDI (early post-engraftment and late post-transplant) were neither previously exposed to antibiotics nor to chemotherapy. All but one of these patients had an immunosuppressive and/or biologic therapy in the month prior to CDI diagnosis. All but two were under antibiotic prophylaxis (mainly trimethoprim/ sulfamethoxazole for *P. jirovecii* prophylaxis).

**Table S4**. Risk factors for CDI recurrence

|  | Univariable  OR (95% CI); P | Multivariable  OR (95% CI); P |
| --- | --- | --- |
| Age | 1.02 (0.98 – 1.05); 0.41 | NS |
| Healthcare related CDI | 3.91 (0.87 – 17.89); 0.08 | NS |
| Number of antibiotics prior to CDI diagnosis | 1.41 (1.07 – 1.86); 0.01 | 1.54 (1.12-2.12); 0.008 |
| Cephalosporin use prior to CDI diagnosis | 3.00 (1.1 – 8.0); 0.03 | NS |
| Immunosuppressive therapy | 7.80 (1.00 – 60.96); 0.05 | 9.69 (1.06-88.45); 0.04 |
| Malignancy other than acute leukemia | 3.85 (1.46 – 10.16); 0.006 | 4.85 (1.64-14.32); 0.004 |

OR: odds ratio; CI: confidence interval; NS: non-significant.

The same results were obtained by stepwise logistic regression using stepwise forward and backward selection, as well as Akaike Information Criterion (AIC).
